# How Stem and Progenitor Cells Can Affect Renal Diseases

**DOI:** 10.3390/cells13171460

**Published:** 2024-08-30

**Authors:** Francesca Montenegro, Francesca Giannuzzi, Angela Picerno, Antonella Cicirelli, Emma Diletta Stea, Vincenzo Di Leo, Fabio Sallustio

**Affiliations:** 1 Department of Interdisciplinary Medicine, University of Bari Aldo Moro, 70124 Bari, Italy; francesca.montenegro@uniba.it (F.M.); francesca.giannuzzi@uniba.it (F.G.); angela.picerno@uniba.it (A.P.); antonella.cicirelli@uniba.it (A.C.); dileovincenzo88@gmail.com (V.D.L.); 2 Department of Precision and Regenerative Medicine and Ionian Area, University of Bari Aldo Moro, 70124 Bari, Italy; emmadiletta.stea@gmail.com

**Keywords:** stem-cell transplant, renal progenitors, regenerative mechanisms, IgA nephropathy, diabetic nephropathy, C3 glomerulopathy, focal segmental glomerulosclerosis, idiopathic membranous nephropathy, anti-glomerular basement membrane glomerulonephritis, ANCA-associated crescentic glomerulonephritis

## Abstract

Stem and progenitor cells have been observed to contribute to regenerative processes in acute renal failure and chronic kidney disease. Recent research has delved into the intricate mechanisms by which stem and progenitor cells exert their influence on kidney diseases. Understanding how these cells integrate with the existing renal architecture and their response to injury could pave the way for innovative treatment strategies aimed at promoting kidney repair and regeneration. Overall, the role of stem and progenitor cells in kidney diseases is multifaceted, with their ability to contribute to tissue regeneration, immune modulation, and the maintenance of renal homeostasis. Here, we review the studies that we have available today about the involvement of stem and progenitor cells both in regenerative therapies and in the causes of renal diseases, as well as in natural healing mechanisms, taking into account the main kidney disorders, such as IgA nephropathy, lupus nephritis, diabetic nephropathy, C3 glomerulopathy, focal segmental glomerulosclerosis, idiopathic membranous nephropathy, anti-glomerular basement membrane glomerulonephritis, and ANCA-associated crescentic glomerulonephritis. Moreover, based on the comprehensive data available in the framework of the specific kidney diseases on stem cells and renal progenitors, we hypothesize a possible role of adult renal progenitors in exacerbating or recovering the illness.

## 1. Introduction

Kidney diseases have increasingly become a major health concern worldwide, carrying significant implications for patient morbidity and mortality. Understanding the role of stem and progenitor cells in kidney diseases is critical, as they play a significant role in the development, progression, and potential regeneration of damaged renal tissue [1].

Stem and progenitor cells have been observed to contribute to regenerative processes in acute renal failure and chronic kidney disease [2,3,4,5,6]. Recent research has delved into the intricate mechanisms by which stem and progenitor cells exert their influence on kidney diseases. These specialized cells have been found to not only participate in renal tissue regeneration but also to modulate the immune response and promote tissue repair. Moreover, the interplay between stem and progenitor cells and the microenvironment within the kidney has been a topic of intense investigation, shedding light on the complex signaling pathways and cellular interactions involved in renal disease pathology [5,6,7,8,9].

Furthermore, the potential of harnessing the regenerative capabilities of stem and progenitor cells for therapeutic interventions in kidney diseases has garnered considerable interest [10,11]. Understanding how these cells integrate with the existing renal architecture and their response to injury could pave the way for innovative treatment strategies promoting kidney repair and regeneration. Overall, the role of stem and progenitor cells in kidney diseases is multifaceted, with their ability to contribute to tissue regeneration, immune modulation, and the maintenance of renal homeostasis [12,13]. Continued research in this field holds promise for unveiling novel therapeutic approaches to address the growing burden of kidney diseases. This knowledge can provide a foundation for the development of novel therapeutic strategies that target these cells and promote kidney repair. Furthermore, understanding the involvement of stem and progenitor cells in kidney diseases can also aid in identifying the early biomarkers and critical genes that contribute to disease susceptibility and progression [13]. In this context, animal models play a crucial role in studying the involvement of stem and progenitor cells in kidney diseases. These models allow researchers to study the behavior and function of these cells in a controlled environment, providing valuable information about their role in kidney regeneration and damage.

Here, we review the data we have available today about the involvement of stem and progenitor cells both in regenerative therapies, in causing renal diseases, and in natural healing mechanisms, taking into account the main kidney disorders, such as IgA nephropathy, lupus nephritis, diabetic nephropathy, C3 glomerulopathy, focal segmental glomerulosclerosis, idiopathic membranous nephropathy, anti-glomerular basement membrane glomerulonephritis, and ANCA-associated crescentic glomerulonephritis.

Moreover, based on the comprehensive data available in the framework of the specific kidney diseases on stem cells and renal progenitors, we hypothesize a possible role of adult renal progenitors in exacerbating or recovering from the illness.

## 2. IgA Nephropathy and Stem Cells

Immunoglobulin A nephropathy (IgAN) is one of the main causes of glomerulonephritis and kidney failure [14]. Although the etiopathogenesis of IgAN is not yet completely clear, a four-hit process supporting the onset of the disease has been described and widely accepted, most notably the abnormal production of galactose-deficient IgA1 (gd-IgA1), whose circulating levels are higher than in healthy subjects. The presence of anomalous gd-IgA1 triggers the massive production of IgG autoantibodies, which recognize de-glycosylated IgA1 and form IgA1-IgG immune complexes that are not efficiently cleared from circulation and terminally precipitate in the glomerular mesangium [14,15,16].

Multiple factors have been identified as decisive in triggering the onset of the disease, such as genetic predisposition, viral or microbic infections, and environmental exposition [17,18,19,20,21]. However, it is indisputable that immune-system dysregulation has a role in the pathogenesis of IgAN, both regarding B cells and T cells. In fact, an increased number of IgA-producing B cells, whose activation may be CD4 T-cell dependent or independent and a dysfunction in the lymphocytes compartment, has been observed in IgAN, such as those regarding Th1/Th2 imbalance, T reg activity, and T follicular helper [15,22]. These cells play a role in the adaptative and innate immune response via direct cell–cell interactions, as well as by cytokines, growth factors, and chemokines. Innate immunity also cannot be neglected, as its activation mediated by Toll-like receptors (TLRs) may drive glomerular loss of function, exacerbating IgAN [23].

T cells, B cells, Kupffer cells, and mucosal intraepithelial cells are bone-marrow-derived cells known to be involved in IgAN. So, during the last few decades, IgAN has somehow been identified as a bone-marrow stem-cell disorder. These conclusions were reached once IgAN mice undergoing bone-marrow transplantation showed attenuated mesangial IgA and C3 deposition, reduced IgA level, and macromolecular size in the serum, demonstrating that cells derived from bone marrow may contribute to determining qualitative and quantitative changes of serum IgA [24]. Similarly, patients with both acute myeloid leukemia and IgA nephropathy showed complete remission for IgAN after bone-marrow transplantation [25].

Moreover, a recent clinical case demonstrated that strong bone-marrow stimulation by granulocyte colony-stimulating factor (G-CSF) caused a highly active form of IgA nephropathy, diagnosed following immunofluorescence on a renal biopsy specimen that showed massive staining of the mesangium for IgA light chains, C3, and lambda [26]. Although the exact molecular mechanisms are unknown, these data suggest that a malfunction of the hematopoietic stem cells (HSCs) in the bone marrow may play a role in the onset and maintenance of the pathological phenotype of IgA nephropathy.

In addition to their presumed role in driving the onset of IgAN, stem cells could represent an interesting alternative in the therapeutic treatment of IgA nephropathy. Indeed, mesenchymal stem cells (MSCs) exhibit multiple properties, including proliferative, tissue damage repair, and immunological features [27]. MSCs can be isolated from bone marrow, as well as from adipose tissue (adipose tissue-derived stem cells, ADSCs). An autologous preparation of ADSCs can easily be obtained, and this is an advantage.

**Figure 1:**
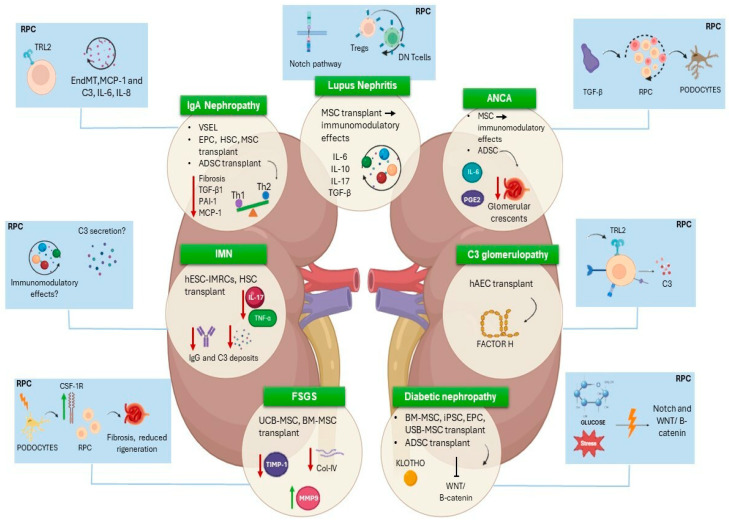
**IgA nephropathy (IgAN) and stem cells (SC).** VSELs can potentially repair kidney damage in IgAN. EPCs improve renal function and slow disease progression in IgAN. HSCs may influence IgA production and IgAN pathology. MSCs, including ADSCs, can reduce fibrosis, inflammatory molecules such as TGF-β1, PAI-1, and MCP-1, and Th1/Th2 cytokine balance. **IgAN and renal progenitors cells (RPC).** TLR2 plays a role in the activation of RPC via autocrine signaling; its stimulation leads to the modulation of EndMT and the secretion of inflammatory mediators such as MCP-1, IL-6, IL-8, and C3. **Lupus nephritis (LN) and SC**. MSC have immunomodulatory effects on various immune cells involved in the pathogenesis and can modulate the secretion of cytokines such as IL-10, IL-17, and TGF-β. **Lupus nephritis and RPC**. Promoting notch activation may enhance renal progenitor proliferation during the regenerative phases of glomerular injury, potentially aiding tissue repair and regeneration. RPCs have immunomodulatory capacity and exhibit immunomodulatory properties by influencing regulatory (Tregs) and double-negative (DN) T cells. **Anti-glomerular basement membrane glomerulonephritis, ANCA-associated Crescentic glomerulonephritis, and SC.** MSCs have anti-inflammatory, proliferative, immunomodulatory, and antioxidant effects. ADSCs reduce the recruitment of immune cells, such as CD8+ T cells and CD68+ macrophages, and release factors such as PGE2 and IL-6, which promote a reduction in the formation of glomerular crescents. **Anti-GBM, ANCA-associated crescentic glomerulonephritis, and RPC.** TGF-β drives the clonal expansion of a single RPC clone, inducing them to produce extracellular matrix. **C3 glomerulopathy (C3G) and SC**. The hAEC transplant into the liver of neonatal mice deficient in complement Factor H resulted in the production of a sufficient amount of human FH, which was useful in preventing complement activation and glomerular deposition of C3 in C3 glomerulopathy. **C3G and RPC.** Following TLR2 stimulation by pathogen-associated molecular patterns, the RPCs express high local levels of C3, which can contribute to renal injury and fibrosis. **Diabetic nephropathy (DN) and SC.** BM-MSCs, UCM-MSCs, iPSCs, and EPCs have been experimented on in DN. ADSC transplantation activates Klotho and inhibits WNT/ β-catenin. **DN and RPC.** In conditions of chronic hyperglycemia and oxidative stress, the regenerative response and notch and WNT/β-catenin signaling pathways can be compromised. **Focal segmental glomerulosclerosis (FSGS) and SC.** UCB-MSC transplantation significantly reduces urinary protein and serum creatinine levels, delays glomerular sclerosis, and improves podocyte and basement membrane recovery. Transplantation of BM-MSCs downregulates TIMP-1, upregulates MMP9, and reduces extracellular matrix accumulation in the renal tissues of rats with FSGS. **FSGS and RPC**. Damage to podocytes upregulates CSF-1R in RPCs, leading to the development of a profibrotic phenotype and reduced regeneration. **Idiopathic membranous nephropathy (IMN) and SC**. The administration of hESC-IMRCs in rat models of MN reduced proteinuria and improved renal histology by modulating immune responses, enhancing IL-10 production, and reducing IL-17, TNF-α, and IgG and C3 deposits. **IMN and RPC.** It could be hypothesized that RPC may affect IMN by C3 secretion and immunomodulatory effects.

In the context of IgA nephropathy, very promising results were observed in mice because ADSCs isolated from different stages of IgAN mice provided similar effects on the kidney [28]. After their isolation from the mice’s inguinal fat pads, cellular *in vitro* expansion and tail-vein re-injection, mesangial proliferation, and glomerulosclerosis were dramatically reduced in ADSC-treated groups. ADSCs strongly reduced fibrotic and inflammatory molecules too, like TGF-β1, PAI-1, and monocyte chemotactic protein-1 (MCP-1), exerting immunomodulating effects. In addition, the treatment affected the Th1/Th2 cytokine balance. It reduced Th1 cytokine activity in the kidney and caused a shift to Th2 responses in spleen T cells (Figure 1) [28].

Interestingly, human ADSCs have quite similar immunoregulatory properties to murine ADSCs, so these results support their use as a therapeutic tool for IgAN.

They were used in the first clinical trial for the treatment of refractory IgA nephropathy to evaluate its safety and tolerability [29]. While corticosteroids represent the main treatment for IgA nephropathy with a high risk of progression, we miss efficient regimens for patients resistant to steroid therapy. The use of ADSC is particularly interesting in clinics because, in addition to their immunosuppressive properties mediated by the release of cytokines and direct actions on T cells, MSCs are poorly immunogenic, lacking MHC-class II costimulatory molecules, unlike other types of stem cells [30]. Therefore, MSC’s immunosuppressive properties may be a strong therapeutic support for IgA nephropathy, especially in refractory IgAN, where ordinary treatments are unsuccessful.

IgAN MSCs are not the only studied stem cells. Flow cytometry studies on whole blood showed an increased level of circulating very small embryonic-like stem cells (VSELs) in IgAN patients compared to controls, while no differences were observed in hematopoietic stem cells (HSCs) or endothelial progenitor cells (EPCs) [31]. 

Physiologically in a dormant state, VSELs represent the remnants of embryonic development. VSELs were identified about twenty years ago in mice bone marrow as a very rare cell population with reduced dimensions (smaller than erythrocytes), positive for stem-cell antigen-1 (Sca-1) but negative for hematopoietic lineage markers Lin and CD45 [32,33]. Mice VESLs express SSEA-1, Oct-4, Nanog, Rex-1, Dppa3, and Rif-1, while human VESLs show SSEA-4, Oct-4, and Nanog, as well as CD133 [32,34]. These cells have been studied for a long time, and they have also been identified in specific cell niches in different adult tissues [33,35]. Bone-marrow-resident VSELs can be activated and mobilized into peripheral blood in case of tissue damage, supporting different tissue regeneration [36,37,38]. Chronic inflammation may represent the cause of the increased presence of the VSELs subset in patients’ whole blood, and it could be responsible for an attempt to repair tissue damage by exploiting the differentiation capacities of VSELs. Therefore, these stem-cell populations could be an interesting tool to be studied and triggered in order to repair kidney damage in IgAN patients. Lastly, endothelial progenitor cells (EPCs) have been explored as a therapeutic option in IgAN, showing promise in improving renal function and slowing disease progression in rat models [39]. EPCs share a number of cell surface markers with vascular endothelial cells and can differentiate into endothelial cells, contributing to the formation of new blood vessels. Guo et al. showed that EPC transplantation in IgAN rats may delay glomerular sclerosis progression in IgAN, promote angiogenesis, and increase peritubular capillary (PTC) density. Moreover, EPCs down-regulated HIF-1a, MCP-1 expression, and improved hypoxia, and inflammation [39].

Collectively, these findings underscore the multifaceted potential of various stem-cell types in treating IgAN, offering new avenues for regenerative therapies that could overcome the limitations of traditional treatments and improve patient outcomes. However, except for studies on the therapeutic potential, there are still no studies focusing on stem-cell involvement in molecular mechanisms of IgAN, although they could contribute to a further understanding of the disease.

## 3. IgA Nephropathy and Renal Progenitors

Renal progenitors, which are crucial for maintaining kidney integrity and regenerative capacity, may also play a role in the development and progression of IgA nephropathy (IgAN). These progenitor cells contribute to kidney homeostasis and repair by differentiating into various renal cell types, including podocytes and tubular cells [5,8,40].

In the human kidney, human adult renal stem/progenitor cells (RPCs) are CD24^+^/CD133^+^ cells located in the glomeruli and the proximal tubules of nephrons, exhibiting epithelial expansion, self-renewal, and differentiation capacities. Moreover, these cells also express the embryonic renal factor PAX2. RPCs have demonstrated the ability to regenerate extensive portions of renal tubules and replace lost podocytes in cortical nephrons, thus representing a highly promising cell population with substantial potential for developing future therapies for both acute and chronic renal injuries [41].

In the context of IgAN, a disease characterized by glomerulosclerosis and interstitial fibrosis, the regenerative potential of renal progenitors could be pivotal. As previously discussed, studies have shown that IgAN patients exhibit selective mobilization of very small embryonic-like stem cells (VSELs) and increased maturation of monocytes towards an M2-like and angiopoietic phenotype, suggesting an active, but possibly insufficient, regenerative response [31].

The role of renal progenitors in IgAN is further supported by evidence that altered growth or differentiation of these cells can impact kidney regeneration, as seen in other renal disorders like diabetic nephropathy [42]. Additionally, the identification of renal progenitors from the urine samples of patients with kidney disorders, including those with genetic mutations affecting podocyte proteins, underscores their potential in personalized medicine and disease modeling [43,44].

Interestingly, a recent study showed that the expression of CD31, a marker of endothelial cells, decreased significantly in IgAN patients, while the expression of α-smooth muscle actin (α-SMA) and vimentin, markers associated with mesenchymal cells, increased significantly. This suggests a potential endothelial–mesenchymal transition (EndMT) in the pathogenesis of renal fibrosis in IgAN [45]. Renal stem/progenitor cells have also the capacity to control the EndMT process through the secretion of antiseptic peptides, such as CXCL6, SAA4, and BPIFA2 [46], and, therefore, may contribute to regulating renal fibrosis in IgAN. Moreover, the stimulation of TLR2 in adult renal progenitor cells (RPCs) led to the secretion of inflammatory mediators like MCP-1, IL-6, IL-8, and C3 through NF-kB activation. This activation also increased RPC proliferation rates, suggesting a role for TLR2 in RPC activation via autocrine signaling (Figure 1) [41]. Therefore, C3 deposits found in the IgAN glomeruli may be the trail of an attempt to repair the damage by RPCs or can be exacerbated by the RPC inflammatory response.

While the exact mechanisms by which renal progenitors influence IgAN remain to be fully elucidated, their involvement in kidney repair and regeneration highlights their potential as targets for innovative treatment strategies. Thus, renal progenitors represent a promising avenue for understanding and potentially mitigating the pathogenesis of IgAN.

## 4. Lupus Nephritis and Stem Cells

Lupus nephritis (LN) is a major complication of systemic lupus erythematosus (SLE), which affects up to 60% of SLE patients, leading to a significant increase in the morbidity and mortality rate [47]. A key characteristic of kidney damage is the build-up of immune complexes (ICs) in the glomeruli, resulting in kidney inflammation, injury, and scarring. Despite significant improvement in the treatment of LN over the past 50 years, clinical remission is only achieved in half of the patients, and significant side effects have been described [48]. Conversely, MSCs have shown promising results in improving disease activity and renal function in LN patients [49].

Studies have highlighted the immunomodulatory effects of MSCs on various immune cells involved in LN pathogenesis, such as T cells, macrophages, and plasma cells [50,51]. Additionally, the regenerative potential of stem cells, particularly MSCs, in protecting and repairing injured kidney cells has been emphasized, along with their anti-inflammatory actions that can counteract the mechanisms of glomerulonephritis [2]. Immunohistochemical analysis using CD34 expression has been proposed as a potential surrogate marker for disease activity in LN patients, indicating a correlation between interstitial stem cells and disease severity [52].

Stem cells, particularly MSCs, play a crucial role in regenerating damaged kidneys in LN by exerting immunomodulatory effects and promoting tissue repair. MSC therapy has been shown to reduce pro-inflammatory immune cells, like central memory CD4+ T cells and cytotoxic tissue-resident memory CD8+ T cells, while increasing anti-inflammatory T-cell subtypes and regulatory T cells [50,51]. MSC therapy in LN was investigated using single-cell sequencing. MSC treatment led to a reduction in pro-inflammatory central memory CD4+ T cells (Tcm), cytotoxic tissue-resident memory CD8+ T cells (Trm), and exhausted CD8+ T cells, while increasing anti-inflammatory naive/effector CD8+ T cells and type-1 regulatory T cells (Tr1). The effects of MSC therapy extended to immune cells in kidney regional immunity in LN. MSC treatment reduced infiltrating pro-inflammatory Ly6c hi/inter/lo era2+ macrophages and increased anti-inflammatory resident macrophages and Ly6c lo ear2− macrophages.

Furthermore, MSC therapy decreased long-lived plasma cells, pro-inflammatory neutrophils, and dendritic cells in the kidney’s regional immunity in LN patients [50]. Overall, the study findings suggest that MSC therapy modulates the immune-cell profile in the kidney’s regional immunity, favoring an anti-inflammatory environment and laying a foundation for potential clinical applications in LN treatment.

In lupus mice, as well as in patients with LN, MSCs demonstrate their immunomodulatory effects through the suppression of autoimmunity, enhancement of renal pathology, and restoration of kidney function [51]. It has been observed that MSCs derived from human embryonic stem cells possess the ability to decrease the levels of serum IL-6 in mice prone to lupus and can potentially hinder or decelerate the progression of lupus-related glomerular disease. The regulatory role of MSCs extends to the modulation of the secretion of crucial cytokines, such as IL-10, IL-17, TGF- β, tumor necrosis factor-alpha (TNF-α), granulocyte–macrophage colony-stimulating factor (GM-CSF), and high mobility group box 1 protein (HMGB-1), all of which play significant roles in the development of LN [51] (Figure 1).

Additionally, MSCs can protect and regenerate injured kidney cells, including tubular cells, podocytes, and endothelial cells, through their multi-lineage differentiation ability and anti-inflammatory actions. As a result of tissue damage and the secretion of inflammatory molecules, chemokines, adhesion molecules, and matrix metalloproteinases, MSCs are able to migrate to damaged sites, such as renal tissue, and can engraft, differentiating into mesangial cells and allowing renal function to resume [53]. Studies have shown that MSC transplantation with high-dose immunosuppressive therapy significantly decreases disease activity, proteinuria, and autoantibody levels in SLE patients with LN, highlighting the efficacy and safety of this approach in treating LN [49].

Interestingly, mesenchymal stromal-like cells (MSLCs) were detected in the kidneys of young lupus-prone mice and within the developed tertiary lymphoid structures (TLS). In young mice, MSLCs were mainly located in the pelvic wall, while in older mice with proteinuria, MSLCs were found around and within the TLS. MSLCs were observed adjacent to CD45 immune cells, indicating their potential role in the organization of TLS. Stimulation of human mesenchymal stem cells (MSCs) with pro-inflammatory cytokines TNF-α and IL-1β led to increased expression of various genes involved in inflammation. The stimulated MSCs induced the proliferation of CD4+ T cells, but a suppressive effect was noted when co-cultured with non-stimulated MSCs. Co-culture of stimulated MSCs with T cells from healthy donors resulted in a contact-dependent increase in Th2 and Th17 subsets, suggesting a role for MSCs in modulating T-cell responses [54]. The study suggests that tissue-specific or migratory MSCs may act as lymphoid tissue organizer (LTo) cells, accelerating early inflammatory processes and initiating the formation of kidney-specific TLS in chronic inflammatory conditions.

MSCs have shown promise in modulating immune responses in conditions such as lupus and LN. However, research indicates that the effectiveness of MSCs in regulating the immune system can differ from person to person. Furthermore, the long-term effects and safety of using MSCs for immune modulation in these conditions are still being investigated and remain incompletely understood. Despite the promising results, the efficacy of MSC therapy in LN requires further validation in clinical settings, and future research is needed to identify the most potent MSC subpopulations and optimize treatment protocols. Overall, MSCs represent a multifaceted therapeutic approach that could overcome the limitations of current LN treatments, offering hope for improved patient outcomes.

## 5. Lupus Nephritis and Renal Progenitors

Renal progenitors may play a crucial role in the development of LN by contributing to the pathogenesis through various mechanisms. Research has shown that, in patients with refractory lupus nephritis (RLN), functional genetics on patient-derived podocytes confirmed lupus podocytopathy as a cause of RLN, indicating the involvement of renal progenitor cells in LN development. Indeed, patient-derived renal progenitors were differentiated into podocytes, revealing aberrant characteristics such as abnormal nephrin trafficking, cytoskeletal structure, lysosomal leakage, and increased detachment compared to controls, confirming lupus podocytopathy as a cause of RLN [55].

As previously described, the pathogenesis of LN involves complex interactions between immune cells and kidney-resident cells, including podocytes, mesangial cells, and tubular epithelial cells. Podocytes can present antigens and participate in immune responses, while mesangial cells produce pro-inflammatory cytokines and extracellular matrix, contributing to glomerular fibrosis [56,57,58]. Additionally, tubular epithelial cells modulate the interstitial environment to promote T-cell infiltration and the formation of tertiary lymphoid organs, further exacerbating inflammation and fibrosis [56,59]. The presence of fibrocytes, which are extracellular matrix-producing cells derived from bone-marrow progenitors, has been identified through the detection of cells positive for the expression of collagen type I (colI), α-SMA, CD34 and CD45 but negative for macrophage (CD115)- and fibroblast (CD90)-specific markers. These cells were found in the peripheral blood and in the kidneys of patients with LN, particularly those with interstitial fibrosis. These fibrocytes, mainly located in the tubulointerstitium, were significantly higher in patients with proliferative LN than in those with non-proliferative LN. Moreover, significant differences in the numbers of fibrocytes were observed in the presence of chronic pathological conditions, such as interstitial fibrosis, tubular atrophy, glomerular crescents, and glomerular sclerosis. A negative correlation was found in the number of these cells with estimated glomerular filtration rate (eGFR) levels [58]. The involvement of various immune cells, including T cells, B cells, macrophages, and dendritic cells, further complicates the pathogenesis of LN. These cells produce cytokines, such as IL-12 and IL-18, which attract other inflammatory cells and maintain kidney inflammation, leading to glomerular damage [57].

For these reasons, RPCs can be exploited in LN for two different properties. First, they can regenerate podocytes [60] and may be stimulated to enhance this capacity [61]. Manipulating this balance could potentially trigger podocyte regeneration in patients with glomerular disorders, offering a new approach to treatment. By inhibiting the notch pathway, interventions could be designed to ameliorate proteinuria and prevent podocyte loss during the early stages of glomerular injury. Conversely, promoting notch activation may enhance renal progenitor proliferation during the regenerative phases of glomerular injury, potentially aiding in tissue repair and regeneration (Figure 1) [61].

Second, RPCs have an immunomodulatory capacity [62]. They exhibit immunomodulatory properties by influencing specific T-cell subpopulations, such as T regulatory cells (Tregs) and double-negative (DN) T cells (Figure 1). The dynamic changes observed in Tregs and DN T cells during the co-culture experiments suggest a time-dependent modulation of these T-cell subpopulations by activated RPCs [62]. This temporal aspect of immune-cell modulation by RPCs is crucial in the response to diseases such as LN. The ability of RPCs to regulate the balance between immune tolerance and autoimmunity through the modulation of Tregs and DN T cells suggests a potential therapeutic avenue for immune-related disorders. Understanding how RPCs interact with the immune system opens up new possibilities for targeted immunomodulatory therapies.

Overall, the interplay between renal progenitors, immune cells, and resident kidney cells underscores the multifactorial nature of LN and highlights the potential for targeted therapeutic approaches to restore kidney function and mitigate disease progression.

## 6. Diabetic Nephropathy and Stem Cells

Diabetes mellitus, a worldwide health issue, has reached epidemic levels, with an estimated 643 million individuals globally expected to be living with the condition by 2030 [63]. The statistical data indicate that over 150 million diabetes sufferers may develop diabetic kidney disease (DKD) by 2030 [64]. Despite diabetic patients being treated with angiotensin receptor blockers (ARBs), the risk of renal disease progression over a two-year period increases with higher levels of proteinuria and albuminuria, as well as decreased eGFR [65].

In addition to pharmacological and lifestyle modifications, innovative approaches are required to halt DKD progression or regenerate damaged renal tissue, such as cell therapy. In pre-clinical models, it has been observed that stem-cell therapy could be a potential treatment for diabetic nephropathy [66]. In this context, syngeneic, autologous, allogeneic, or xenogeneic mesenchymal stem cells have demonstrated potential applications for the treatment of DKD [67].

MSCs have been widely experimented with in diabetic nephropathy (DN) and have demonstrated the ability to differentiate into renal cells and repair and regenerate damaged tubular and glomerular structures. Hypoxia, inflammation, and hyperglycemia can stimulate the migration and proliferation of MSCs in diabetes. However, hyperglycemia and hyperinsulinemia can hinder MSC function and restrict their potential [68].

The regenerative potential of therapy with MSCs was evaluated also in a study with adriamycin (ADR)-induced diabetic nephropathy rats. MSCs limited podocyte loss and apoptosis, preserved nephrin and CD2AP, and reduced glomerulosclerosis. Furthermore, MSCs exhibited anti-inflammatory effects and decreased RPC dysfunction. These findings suggest that MSCs may represent an effective strategy to preserve podocyte viability and reduce glomerular inflammation and sclerosis [69].

Studies on stem-cell-based therapy for DN have utilized different types of stem cells, such as bone-marrow-derived MSCs (BM-MSCs), urine-derived stem cells (USCs), adipose tissue-derived stem cells, induced pluripotent stem cells (iPSCs), and endothelial progenitor cells (EPCs) [2,70].

BM-MSC injection enhanced pancreatic and renal function, reduced mesangial expansion and thickening of the glomerular basement membrane, mitigated podocyte damage and proteinuria, and inhibited the increase in kidney weight and glomerular hyperfiltration in the early stages of DN [2,71].

The relationship between BM-MSCs and miRNA MiR-124a, which affects the differentiation potential of pancreatic progenitor cells, was also examined. It was observed that BM-MSCs could be induced into islet-like cells, and miR-124a promoted this differentiation. This process involved islet cell-specific transcription factors regulated by miR-124a, apoptosis-related genes, podocyte-related genes, and the activity of the notch signaling pathway [72]. Nevertheless, BM-MSCs combined with miR-124a alleviated renal injury caused by DN and reduced podocyte apoptosis induced by hyperglycemia (HG). The protective effect of BM-MSCs combined with miR-124a was closely related to the inactivation of the notch signaling pathway. Hence, MSCs combined with miR-124a protected renal tissue from deterioration and inhibited nephrocyte apoptosis in DN [72].

Recent studies have confirmed that MSC transplantation can reduce insulin requirements and attenuate inflammation in both the blood and renal tissue. It has also been observed that MSCs improve renal function and histology and reduce the expression of sodium–glucose cotransporter 2 (SGLT2) on renal tubular epithelial cells (RTECs) [73]. By modulating the pro-inflammatory process via the lipoxin A4 LXA4-ALX/FPR2 axis, MSCs decrease the likelihood of DN progressing to glomerulosclerosis, inhibit glomerulosclerosis and pro-inflammatory cytokines, and contribute to kidney homeostasis [74]. Additionally, MSCs exert an anti-fibrotic effect, reducing the accumulation of extracellular matrix caused by DN [75].

In other studies, the transplanted BM-MSCs increase the serum levels of EGF and IL-10, reduce systemic inflammation, and secrete hepatocyte growth factor (HGF) and MCP-1, inhibiting macrophage infiltration and oxidative stress [76]. Notably, the BM-MSC secretome has demonstrated potential clinical applications and includes both soluble proteins (cytokines, chemokines, growth factors, and proteases) and factors released in extracellular vesicles [77].

Increasingly studies have demonstrated that extracellular vesicles (EVs), mainly exosomes and microvesicles, participated in the pathophysiological process of DN. Recently emerging studies have also found that the EV content in urine (miRNA, mRNA, and protein) could be used as potential biomarkers for DN [78].

On the other end, microvesicles derived from MSCs (MSC-derived EVs), confer tissue repair and immunomodulation through a paracrine mechanism [79]. MSC-EVs demonstrated the capacity to preserve tight junctions in renal tubular epithelial cells and exhibited an anti-apoptotic effect comparable to that of MSCs. Additionally, MSC-EVs appeared to induce autophagy by inhibiting the mTOR signaling pathway, thereby attenuating the expression of fibrosis markers and histologic damage in a diabetic nephropathy rat model. Specific exosomal miRNAs that account for the nephroprotective roles of MSCs have been identified. For example, miRNA-let-7a carried by BM-MSC-EVs suppressed renal cell apoptosis [79].

Research using stem cells from various origins may present an intriguing therapeutic potential in diabetic nephropathy (DN), as these cell types might release extracellular vesicles with unique contents and properties.

Another kind of MSC involved in DN therapy is ADSC, which showed, in different models of DN, advantageous effects not only in the early stages but also in advanced DN. Streptozocin-DN rats treated with ADSCs activated Klotho and inhibited WNT/β-catenin, oxidative stress, and p38-MAPK signaling pathways (Figure 1) [80]. Klotho is downregulated in different models of CKD. Its loss is associated with WNT/*β*-catenin signaling activation, since it physically binds to multiple Wnt ligands, including Wnt1, Wnt3a, Wnt4, and Wnt7a, acting as an endogenous antagonist of WNT/*β*-catenin signaling. Anyway, its upregulation exerts kidney-specific protective effects against renal disease and complications of chronic kidney disease. Therefore, ADSCs may represent an interesting tool to restore high levels of Klotho [80,81].

Moreover, ADSCs-derived EVs can re-establish synaptic peptides and renin in podocytes, both by transporting EGF and miRNA cargo, including also the MiR-26a-5p, and they can inhibit apoptosis and deactivate NF-κB by targeting TLR4 in renal cells [82,83].

Umbilical cord blood-derived mesenchymal stromal cells (UCB-MSCs) can mitigate DN by acting on hyperglycemia-activated podocytes via TLR 2 and TLR 4 signaling pathways, as well as ameliorate the activation of proinflammatory cytokines released by podocytes [84]. In fact, once co-cultured with podocytes treated with high glucose or transplanted in diabetic mice, UCB-MSCs decreased the expression of the endogenous ligands of TLRs, such as HSP70 and HMGB1, as well as the level of TLR2 and TLR4 and the downstream protein level of MyD-88 and P65. Furthermore, the expression of inflammatory cytokines, such as IL-6, IL-β, TNF-α, and MCP-1, was decreased. These results indicated that UCB-MSCs decreased the inflammation and restrained the TLR signaling pathway. They can also reduce the mRNA expression of TGF-β1, α-SMA, collagen I, and heat shock protein-47 and increase the expression of E-cadherin and BMP-7 [84]. Moreover, conditioned medium from mouse UCB-MSCs decreased fibronectin and collagen I deposition both by inhibiting TGF-triggered myofibroblast transdifferentiation and cell proliferation on one hand and by increasing matrix metalloproteinase levels [85]. Additionally, the UCB-MSCs secretome might be comparable to the BM-MSCs secretome’s richness of angiogenic factors [86].

Furthermore, reduced or non-functional levels of circulating EPCs have been found in diabetes and uremia. Among the possible consequences, there is diabetic nephropathy. Notably, various medications can mobilize EPCs from bone marrow (BM) to the sites of vascular injury. Insulin, statins like atorvastatin, and recombinant human erythropoietin can all increase circulating EPCs and boost their therapeutic potential for microvascular damage [2,87].

Another kind of stem cells experimented with in DN therapy is iPSCs [88]. iPSC-MSCs can be generated through clonal expansion and differentiated into various cell types, including osteoblasts, adipocytes, and chondrocytes, promoting tissue regeneration. They also exhibit reduced senescence due to higher telomerase activity compared to BM-MSCs, which can contribute to a decreased likelihood of potency loss during long-term MSC culture [88]. Additionally, iPSC-MSCs have the advantage of being derived from autologous cells, making them more suitable for therapies in personalized medicine. Several strategies have been explored to develop podocytes from iPSCs [66]. Podocytes are specialized cells that filter blood in the glomerulus, and in diabetic nephropathy, they are impaired due to high glucose levels. Therefore, *in vitro* culture of podocytes is critical for the rejuvenation of damaged tissues. Moreover, iPSCs were derived from normal human mesangial cells and exfoliated tubular cells collected from the urine of healthy donors, paving the way for the use of tissue-specific iPSC therapy for renal disorders. These kidney-derived iPSCs can differentiate more effectively into mature renal cells compared to iPSCs from unrelated tissues, with the ability to differentiate into podocyte-like cells while maintaining their proliferative capacity [89,90]. Despite these promising results, significant knowledge gaps remain, particularly regarding the optimal source, dose, and timing of MSC delivery, as well as the need for more clinical trials to confirm their efficacy in humans. Additionally, the use of nanomembrane concentration technology has facilitated the study of protein expression changes in DN, providing new insights into the mechanisms of stem-cell-mediated tissue repair [91]. Overall, while stem-cell therapies, particularly those involving MSCs and their exosomes, offer a promising avenue for treating DN, further research is needed to fully understand their mechanisms and to optimize their clinical application. Indeed, despite these promising findings from preclinical studies, the translation of MSC therapy to clinical practice faces several challenges, including the need to determine the optimal source, dose, and timing of MSC delivery, as well as the development of reliable biomarkers to monitor therapeutic efficacy.

## 7. Diabetic Nephropathy and Renal Progenitors

Another category of stem cells applied to DN in several studies is urine-derived stem cells. Although in not all of these studies, the cells have been fully characterized, it is very likely that they are renal progenitors. Their isolation procedure is simple, non-invasive, and cost-effective, although it can sometimes be time-consuming. In addition to less renal fibrosis, histological damage, and cell proliferation, DN mice administered with USCs also displayed less function loss, cell infiltration, and oxidative stress. Through a paracrine impact, USC can protect renal function by reducing renal interstitial fibrosis, reducing inflammation and oxidative stress, and improving renal tissue structure [92]. It has been observed that EVs derived from USCs can carry important factors such as TGF-β1, angiopoietin, and BMP7, which can reduce hyperglycemia-induced podocyte apoptosis *in vitro*. In another study, the overexpression of miR-16-5p in USC-derived EVs provided protection against hyperglycemia-induced podocyte injury by inhibiting apoptosis [93,94].

In a study on 31 human renal biopsies of DN patients, glomerular RPCs were identified and quantified. The authors found that podocytes progressively reduced, whereas RPCs were still present in advanced DN cases, suggesting an attempt of RPCs to regenerate podocytes in DN [95].

Therefore, regenerative medicine strategies using RPCs can offer a promising alternative to kidney donor shortages [96]. Scientists discovered a novel method for successfully forming RPCs from human urine-derived cells (UCs) that can undergo long-term expansion in a serum-free condition (induced RPCs, iRPCs) [96,97]. Moreover, in the DN animal model, iRPCs demonstrated therapeutic effects, including reduced glomerular hypertrophy and tubulointerstitial fibrosis, lower blood urea nitrogen, serum creatinine, and albuminuria levels, decreased inflammation/fibrosis, enhanced renal regeneration, and confirmed safety [97].

In DN, podocyte injury seems to be a pivotal mechanism in driving the progression of diabetic glomerulosclerosis [98,99]. Consequently, resident renal progenitors in Bowman’s capsule proliferate in an attempt to replace the injured podocyte. However, under conditions of chronic hyperglycemia and oxidative stress, this regenerative response can be impaired, and, if regeneration occurs in a dysregulated manner, it can result in hyperplastic glomerular lesions, scarring, and eventually nephron loss (Figure 1) [100]. Therefore, a potential limitation to glomerular regeneration in DN could be constituted by dysregulated renal progenitor cells that can contribute to the development of hyperplastic lesions [100].

The metabolic state of nephron progenitors is also critical, with disruptions in pathways such as pyruvate and proline metabolism leading to premature progenitor exhaustion and accelerated differentiation, which can further impair kidney function in a diabetic condition [42,101]. In diabetic nephropathy, the altered growth or differentiation of these progenitors can exacerbate kidney damage. Renal progenitors undergo metabolic reprogramming that affects nephropathy development, and metabolites play a key role in epigenetic modifications during kidney development. Metabolites and intermediary metabolism contribute to developmental programs and influence epigenetic and epiproteomic modifications during kidney development [101].

The metabolic stress induced by maternal diabetes can also predispose offspring to renal issues, such as impaired nephron progenitor differentiation, highlighting the importance of metabolic pathways in progenitor cell function and kidney development. Nephron progenitors are essential for kidney development, and their impairment can have long-term consequences on kidney function. Indeed, experiments in mice have shown that offspring exposed to maternal diabetes during pregnancy show a reduction in the total number of nephrons compared to controls, as well as an increased expression of key genes involved in nephron progenitors, such as *Six2* and *Cited1* [102].

Moreover, classical signaling pathways, like notch and WNT/β-catenin, along with other molecular mechanisms, such as the renin–angiotensin–aldosterone system and TLR2, are critical in regulating progenitor responses during homeostasis and injury, and their dysregulation can contribute to diabetic nephropathy (Figure 1) [41,103]. Understanding the transcriptional regulatory networks that govern nephron progenitor maintenance and induction is essential for developing therapeutic interventions to attenuate kidney disease progression in diabetic patients [104,105]. Moreover, these mechanisms highlight the potential of targeting renal progenitors and their regulatory pathways as therapeutic strategies to both mitigate the progression of diabetic nephropathy and improve renal outcomes in affected individuals.

## 8. C3 Glomerulopathy and Stem Cells

C3 glomerulopathy (C3G) represents a heterogeneous group of rare, complex, and severe nephropathies characterized by the predominant deposition of complement C3 in renal tissue [106,107].

The dysregulation of the alternative complement pathway (CAP) in both the “fluid phase” and the glomerular microenvironment forms the basis of the disease pathophysiology. This dysregulation leads to hyperactivity of the C3 convertase “amplification loop” and the buildup of C3 breakdown products, and sometimes C5 products, within the glomerulus. These deposits cause inflammation, kidney damage, and functional impairment. The AP dysregulation may be attributed to the presence of autoantibodies that stabilize the C3 and/or C5 convertases (C3Nef; C5Nef) and, in a smaller percentage of patients, to genetic mutations in complement components or complement inhibitors [108,109]. The complexity of complement dysregulation in kidney disease requires ongoing research efforts. Therefore, a deeper understanding of the precise mechanisms by which the complement system contributes to renal pathophysiology is essential.

Despite numerous studies, the specific pathogenesis of C3G remains unclear. Consequently, an optimal therapeutic treatment for C3 glomerulopathy has not yet been established. In some cases, therapeutic strategies aimed at modulating the complement system have shown promising results, demonstrating the ability to improve renal function. However, the effectiveness of these drugs varies significantly from patient to patient, and desired outcomes are not always achieved [110,111,112,113,114]. Finally, C3G has a high level of disease recurrence after kidney transplantation, ranging from 50% to 80% [115,116]. This aspect underscores the urgency of developing new therapeutic strategies and improving our understanding of the underlying mechanisms of the disease to offer more effective and enduring treatments to patients with C3 glomerulopathy.

The use of stem-cell-based therapies has attracted significant interest as potential new therapeutic strategies that can more precisely modulate this condition. In humans, a complete renal recovery was demonstrated for the first time in 2013 in a patient with a combination of membranoproliferative glomerulonephritis–monoclonal-associated (MG-MPGN) and C3 glomerulonephritis (C3GN) secondary to monoclonal gammopathy. In this case, normalization of the complement system was achieved following chemotherapy and bone-marrow stem-cell transplantation [117]. Following the previously mentioned example, studies on patients with monoclonal gammopathy and glomerular disease characterized by isolated C3 deposits without monoclonal immunoglobulin (MIg) deposits have shown that, after autologous stem-cell transplantation (ASCT), patients achieved complete renal remission. At the eight-month follow-up, high-dose Melphalan administration followed by ASCT proved to be a curative treatment option [118]. Current research continues to focus on understanding the genetic and molecular mechanisms underlying C3GN and how the use of stem cells might be an alternative strategy to provide a normal supply of key complement regulators in cases of genetic abnormalities, such as those affecting complement Factor H (FH), the main plasma regulator of the alternative pathway. A preclinical animal model study demonstrated that human amniotic epithelial cells (hAEC) can differentiate into hepatocyte-like cells *in vivo*. Their administration into the livers of neonatal mice deficient in complement FH resulted in the production of a sufficient amount of human FH, which was useful in preventing complement activation and glomerular deposition of C3 (Figure 1) [119]. In conclusion, while stem-cell transplantation has shown promise in specific C3G cases linked to monoclonal gammopathy, comprehensive studies are needed to establish its efficacy and safety as a standard treatment option for C3G. The emerging field of regenerative medicine is rapidly advancing, and studies are creating a promising frontier in the management of C3 glomerulopathy, offering new hopes for improving clinical outcomes in patients affected by this debilitating condition. As the understanding of C3G’s pathophysiology and the development of anti-complement drugs advance, the future holds promise for more effective and targeted treatments, potentially incorporating stem-cell therapy as a cornerstone of management.

## 9. C3 Glomerulopathy and Renal Progenitors

Renal progenitors may play a crucial role in maintaining kidney integrity, homeostasis, and regenerative capacity, which is particularly relevant in the context of C3G. Renal progenitors respond to podocyte injury by triggering a regenerative program to restore damaged kidney function [6,12]. However, their proliferative responses can be inefficient or excessive, leading to chaotic migration and proliferation, which contributes to crescent formation and glomerular scarring, thereby exacerbating the pathology of C3G [11,12,120].

Complement component C3 plays a significant role in this renal disease, with its secretion by various renal cells, including renal progenitors, being a critical aspect of kidney function and disease. RPCs, following TLR2 stimulation by pathogen-associated molecular patterns, such as zymosan or lipotecoic acid and NFk-B activation, have been shown to express high levels of C3, which is crucial for their role in renal regeneration and repair processes (Figure 1) [41]. The synthesis of C3 by renal cells is not limited to progenitors. Glomerular epithelial cells and proximal tubular epithelial cells also produce and secrete C3, which is regulated by proinflammatory cytokines such as IL-1β, TNF-α, and IL-6 [121,122]. This local production of C3 is essential for mediating inflammatory responses and can contribute to renal injury and fibrosis, as seen in various kidney diseases [123].

Therefore, despite the poor prognosis of C3G, with many patients progressing to end-stage renal disease, the study of renal progenitors offers hope for developing innovative treatments. For instance, understanding the molecular mechanisms that direct renal progenitor responses during homeostasis and following injury could lead to pharmacological tools for preventing and treating glomerulosclerosis, a common outcome in C3G.

## 10. Focal Segmental Glomerulosclerosis and Stem Cells

Focal segmental glomerulosclerosis (FSGS) is a complex glomerular disease characterized by segmental scarring of the glomeruli, leading to proteinuria and potential progression to end-stage kidney disease (ESKD). The pathogenesis of the disease is multifaceted, involving various factors such as podocyte injury, capillary hypertension, and the dysregulation of signaling molecules like transforming growth factor beta and angiotensin [124]. Traditional therapies for FSGS include corticosteroids and immunosuppressants like calcineurin inhibitors and Rituximab, but these treatments often have limited efficacy and significant side effects, particularly in steroid-resistant or dependent cases [125]. Recent advancements have explored the potential of stem-cell therapy as a novel treatment approach. MSCs, including those derived from bone marrow and the umbilical cord, have shown promise in preclinical models by reducing proteinuria, improving renal function, and ameliorating histological damage through their immunomodulatory and reparative properties [126,127]. For instance, UCB-MSCs transplantation in a novel FSGS mouse model significantly decreased urinary protein and serum creatinine levels, delayed glomerular sclerosis, and enhanced podocyte and basement membrane recovery [127]. Similarly, BM-MSCs transplantation in a rat model of FSGS reduced proteinuria, serum creatinine, and urea nitrogen levels, indicating improved kidney function. Mechanistically, BM-MSC transplantation downregulated *TIMP-1* and upregulated *MMP9*, resulting in an increased renal MMP9/TIMP-1 ratio, which may contribute to the attenuation of FSGS progression (Figure 1). BM-MSC transplantation also downregulated the proinflammatory cytokines IL-6 and TNF-α, suggesting an anti-inflammatory effect of BM-MSCs in FSGS treatment [126].

In a study investigating the role of calycosin and BM-MSCs in combating podocyte apoptosis in adriamycin-induced FSGS, CA-pretreated MSCs enhanced the protective effect against podocyte injury and inhibited podocyte apoptosis more significantly than MSCs or CA alone, indicating a synergistic effect that was unexpected [128]. BM-MSCs effectively reduced extracellular matrix accumulation in the renal tissues of rats with FSGS, as evidenced by a lower expression of Col-IV and FN proteins and mRNA in the control group, transplantation group, and positive control group as compared to the model group and agonist group. The body weight, kidney weight, and kidney weight index of rats in the control groups were significantly lower than those in the model group and agonist group, indicating a positive impact of BM-MSCs on these parameters. Moreover, the protein expressions of p38MAPK and p-CREB were lower in the control group and transplantation group, suggesting a potential mechanism through which BM-MSCs exert their effects in FSGS [129].

In a prospective study, five patients with refractory FSGS received autologous bone-marrow-derived mononuclear cells (BMDMCs) via arterial infusion and were followed for 270 days. The safety of the approach was confirmed, with a temporary increase in creatinine that subsequently improved. The infusion was deemed safe and maintained stable renal function [130]. In another study, iPSC lines were generated from patients with FSGS and were confirmed to be a valuable tool for disease [131].

However, the implementation of stem-cell therapies for FSGS is not without its challenges. The optimal source, delivery method, and long-term safety of stem-cell-based interventions remain active areas of investigation. As the scientific community continues to unravel the complex mechanisms underlying FSGS and explore the therapeutic potential of stem cells, the hope is that these advancements will ultimately translate into improved outcomes and a better quality of life for patients suffering from this debilitating condition.

## 11. Focal Segmental Glomerulosclerosis and Renal Progenitors

The pathogenesis of FSGS involves podocyte injury and depletion, with RPCs playing a significant role in disease progression through activation and proliferation [132,133]. Very recent studies have explored innovative therapeutic strategies targeting cellular and molecular mechanisms, such as the CSF-1/CSF-1R axis. The colony-stimulating factor-1 receptor axis (CSF-1/CSF-1R) is important in maintaining and modulating certain stem and progenitor cells and in regulating their niches. Its alteration has been described in FSGS, where it may contribute to enhancing the dysfunctional activation of parietal epithelial cells (PECs), a cell population that constitutes the structure of Bowman’s capsule and that includes RPCs, with an effector role in the pathogenesis of the disease. In fact, consistent *CSF-1R* upregulation was found in RPCs and podocytes in response to podocyte injury, leading to the activation of the ERK pathway and the development of a profibrotic phenotype in RPCs (Figure 1) [132]. However, the administration of CSF-1R inhibitors, such as GW2580, showed therapeutic effects in FSGS by blocking RPC activation, reducing CD44 and pERK12 levels, and maintaining the capacity of RPCs to restore podocyte loss. Moreover, it has been described that CSF-1 had a direct effect on PECs and RPCs, promoting a phenotypic shift from an epithelial to a mesenchymal state, expressing markers such as α-SMA and platelet-derived growth factor β (PDGFR-β), which are associated with FSGS [132]. Lastly, CSF-1R pathway activation affected RPC differentiation and migration. It inhibited the progenitor-to-podocyte transition by downregulating genes related to podocyte transition and inducing the expression of *CXCL12*, which limits podocyte–progenitor turnover. Both PECs/RPCs and podocytes exhibited *CSF-1R* overexpression in FSGS, contributing to the pathology. This overexpression is consistent in both human and experimental models of FSGS.

The involvement of PDGF signaling in FSGS has been highlighted by another study, where PDGF-β produced by injured podocytes activates PECs and, therefore, RPCs, driving a profibrotic switch and disease progression. Inhibition of PDGF-β significantly reduces proteinuria and FSGS, suggesting a potential therapeutic target [133].

These new studies suggest that understanding the molecular mechanisms and genetic underpinnings of FSGS, along with the role of renal progenitors and PDGF signaling, is essential for developing targeted therapies to improve patient outcomes.

## 12. Idiopathic Membranous Nephropathy (IMN) and Stem Cells

Idiopathic membranous nephropathy (IMN or primary membranous nephropathy or PMN) is an autoimmune kidney disease where autoantibodies target specific proteins on podocytes, such as the phospholipase A2 receptor (PLA2R), thrombospondin type 1 domain containing 7A (THSD7A), and neural epidermal growth factor-like 1 (NELL-1) [134,135,136]. IMN represents 80% of cases of membranous nephropathy, which is the leading cause of nephrotic syndrome (NS) in white nondiabetic adults, while the remaining 20% are secondary to conditions like lupus [135,136]. The pathogenesis of membranous nephropathy (MN) involves autoimmune reactions in the kidney glomeruli, characterized by granular IgG and complement system component deposits in the glomerular basement membrane (GBM) near the podocytes. This process leads to GBM thickening and damages the glomerular filtration barrier, causing proteinuria. T cells also play a crucial role by supporting B-cell responses and promoting inflammation. The activation of T cells and the altered signaling in B cells contribute significantly to the autoimmune process [135].

Effective management of patients with membranous nephropathy (MN) requires supportive measures aimed at reducing proteinuria and maintaining blood-pressure control. This typically involves the use of angiotensin-converting enzyme (ACE) inhibitors or angiotensin receptor blockers (ARBs), in line with the KDIGO (Kidney Disease: Improving Global Outcomes) guidelines. Additionally, patients should adhere to dietary sodium restrictions and utilize diuretic therapy as needed, depending on the severity of their nephrotic syndrome [136]. Approximately 30% of patients with MN have a poor response to immunosuppressive therapy and may develop end-stage renal disease [137]. For this reason, it may be useful to discover new targeted therapies. Therapies based on stem cells, both pluripotent and adult, and the bioproducts derived from them, such as soluble factors and extracellular vesicles, could represent a new frontier in the treatment and stimulation of repair processes in chronic renal pathologies, such as membranous nephropathy. Regarding the applications of stem cells in the therapeutic field of IMN, little is known in the literature.

Zhou et al. explored the potential of human embryonic stem-cell-derived immunity-and-matrix regulatory cells (hESC-IMRCs) for treating MN [138]. hESC-IMRCs were MSC-like cells derived from human embryonic stem cells with stronger immunomodulatory functions and abilities in regulating extracellular matrix production [139]. The intravenous administration of hESC-IMRCs in rat models of MN reduced proteinuria and improved renal histology by modulating immune responses, particularly enhancing regulatory CD4 +  CD25 + T cells and IL-10 production, and reducing IL-17, TNF-α, and IgG and C3 deposits (Figure 1). The preclinical findings suggest that hESC-IMRCs may offer a novel therapeutic avenue for MN thanks to their enhanced immunoregulatory effect on Treg cells and their promotion of the shift of T lymphocytes from Th1 to Th2, as well as the reduction of inflammatory cytokines [138].

In the context of HSC transplantation, a unique form of MN associated with the novel antigen protocadherin FAT1 has been identified, highlighting the diverse etiologies and potential for targeted therapies in MN [140,141]. These findings underscore the potential of stem-cell-based therapies for treating IMN by addressing the underlying immune dysregulation and promoting renal repair, although further research and clinical trials are necessary to fully understand their efficacy and safety in human patients. A critical point in the use of stem cells as a therapy is the long-term monitoring, which is essential to rule out potential risks, such as cancer and the development of anti-HLA antibodies. Standardized protocols and a deeper understanding of the *in vivo* mechanisms of action of stem cells could help identify the most suitable cellular sources, optimal administration methods, and appropriate dosages. Overall, the integration of insights from stem-cell research and the identification of novel antigens and genetic markers hold promise for advancing the diagnosis, treatment, and management of IMN and related renal conditions.

## 13. Idiopathic Membranous Nephropathy (IMN) and Renal Progenitors

There are no data whatsoever on renal progenitors’ influence in idiopathic membranous nephropathy. The role of renal stem/progenitor cells in nephron regeneration and repair is gaining attention, with studies showing their multipotency and ability to differentiate into various cellular populations, their immunomodulatory capacity, and their ability to secrete the C3 complement factor (Figure 1) [41,42,60,62]. The dysregulation of these properties may be involved in the IMN pathogenesis or, on the contrary, may be exploited for regenerative purposes. Moreover, the integration of renal stem/progenitor cells and differentiated human cells into complex 3D renal structures *in vitro* further underscores the potential application of stem-cell research for treating renal diseases, including IMN [142,143,144]. These advancements highlight the importance of understanding the molecular and cellular mechanisms underlying IMN and the potential of stem-cell therapies for improving renal outcomes.

## 14. Anti-Glomerular Basement Membrane Glomerulonephritis, ANCA-Associated Crescentic Glomerulonephritis, and Stem Cells

Two pathological conditions, anti-glomerular basement membrane (GBM) antibody-associated glomerulonephritis and anti-neutrophil cytoplasmic autoantibody (ANCA)-associated glomerulonephritis, are two etiological categories of glomerulonephritis (GN) [145]. GN is the second most common cause of chronic kidney disease (CKD), a major global health issue that results in significant morbidity and mortality. Particularly, crescentic GN (CGN), a severe and rapidly progressing form of GN, can lead to end-stage renal disease (ESRD) within days to months [146,147].

CGN is characterized by an immune-mediated inflammation and destruction of the renal glomeruli, frequently resulting in irreversible kidney damage. This condition is marked by crescent formation in the Bowman’s space, resulting from increased permeability of the glomerular capillary membrane, infiltration of immune cells, fibrin, and other plasma protein in Bowman’s space, and proliferation of local cells, which produce cellular crescents. Over time, these crescents can become chronic, evolving into fibrocellular or fibrous crescents with increased matrix and collagen [148]. CGN is diagnosed when crescents appear in over 50% of the sampled glomeruli [149]. Anti-GBM glomerulonephritis and ANCA-associated glomerulonephritis often have crescent formation more than other crescentic glomerulopathies like immune complex glomerulonephritis (such as lupus nephritis). Most current treatments for these autoimmune diseases rely on systemic immunosuppression [147,150]. These therapies are not curative and are associated with broad, non-specific suppression of the immune system, leading to severe adverse effects, such as infections, cancer, and cardiovascular complications.

The common form of anti-GBM disease involves polyclonal IgG antibodies (mainly IgG1 and IgG3) targeting the noncollagenous (NC1) domain of the alpha-3 chain of type IV collagen. Immunoglobulin subclasses IgG4 and IgA are also implicated. Other autoantibodies recently identified in anti-GBM are those against Peroxidasin and Laminin-521, suggesting a broader range of targets within the basement membrane [147,150]. Autoreactive T cells also play a role, contributing to alveolar and glomerular injury and enhancing B-cell function and antibody production. Regulatory T cells (CD4+ CD25+) help modulate this autoimmune response, reducing the severity of the disease [150].

Stem cells, particularly MSCs, have shown promise in treating various forms of glomerulonephritis, including those caused by autoimmune reactions like anti-GBM disease. In particular, MSCs home in injured kidney tissue, where they release a spectrum of anti-inflammatory cytokines and chemokines that can modify the injury’s progression. They are thought to promote healing through paracrine mechanisms that exert anti-inflammatory, proliferative, immunomodulatory, and antioxidant effects, leading to tissue repair and cellular regeneration [151]. In 2013, Furuhashi et al. were the first to demonstrate that MSCs could be promising for treating this kind of glomerular damage [152]. Using a rat model that mimics human anti-GBM-GN, they highlighted that ADSCs have superior therapeutic properties compared to BM-MSCs. Specifically, ADSCs cultured under low-serum conditions were shown to reduce the recruitment of immune cells, like CD8+ T cells and CD68+ macrophages. Moreover, they release factors such as PGE2 and IL-6, which promote the phenotypic switching of glomerular macrophages to immunoregulatory CD163+ cells (Figure 1). This process collectively leads to a reduction in the formation of glomerular crescents [152]. Thus, administering low-serum cultured ADSCs could be a promising and practical cell-based alternative therapy to improve the prognosis of patients with anti-GBM-GN. Furthermore, genetically modified MSCs hold significant promise as carriers for therapeutic genes in the treatment of kidney diseases, such as acute ischemic kidney injury, lupus nephritis, and anti-GBM-GN [153,154]. MSCs engineered to express the antioxidant human glutathione S-transferase Mu 2 (h-GSTM2) provide enhanced protection against anti-GBM-GN in mice [153]. These hGSTM2-MSCs were effective in reducing the expression of inflammatory cytokines, increasing the levels of antioxidant catalase and glutathione peroxidase 1 genes, and inhibiting the infiltration of lymphocytes and macrophages in renal tissues. This approach led to significant clinical improvements in animal models, including reduced proteinuria and better renal function [153].

Moreover, a beneficial anti-inflammatory and immunomodulatory effect of MSCs was shown in rats with anti-GBM-GN [151]. Administering human MSCs (hMSCs) led to significant functional and histological improvements, including reduced proteinuria, serum creatinine, and glomerular crescent formation. hMSCs decreased macrophage and CD8+ cell infiltration in the glomeruli, lowered proinflammatory cytokine levels in the renal cortex, and suppressed Th1 and Th17 responses while promoting Th2 and Tregs.

The MSC-derived conditioned media (MSC-CM) also showed reduced anti-GBM-GN and glomerular fibrosis in Wistar-Kyoto rats via anti-inflammatory and immunomodulatory effects, notably increasing IL-10 levels and promoting M2-type macrophage polarization through MCP-1 induction [155]. Therefore, based on experimental findings, MSCs achieve their beneficial effects by modulating immune responses and producing inhibitory cytokines. They have shown potential in mitigating glomerular damage in crescentic GN through immune-system regulation. However, their application is limited by the invasive methods needed for extraction, the time and costs required for cell culture, and the risk of *in vitro* maltransformation [146,156].

Human amniotic epithelial cells may offer an alternative safe and effective therapeutic option, particularly for autoimmune and inflammatory diseases, due to their strong immunosuppressive properties and favorable safety profile. These cells, easily harvested from the placenta, have low immunogenicity and do not encourage tumor formation [146,157,158]. Their beneficial effects are largely due to their paracrine activity, making them well-suited for regenerative medicine applications. Although there are currently no *in vivo* or *in vitro* studies demonstrating the efficacy of hAEC-based cell therapy for CGN, the positive results observed in other cell and animal models of autoimmune diseases with similar biological characteristics to GN suggest that hAECs could be a promising candidate for testing in this area.

If proven effective in pre-clinical models of early and/or late-stage crescentic GN, hAECs could be useful in clinical practice, hopefully reducing patient mortality and the complications associated with current treatments. Even if stem cells, particularly MSCs, have shown promise in preclinical models of autoimmune diseases due to their immunomodulatory properties and the ability to promote tissue repair, further research is needed to fully understand the mechanisms underlying stem-cell therapy and to establish its safety and efficacy in clinical settings. The integration of stem-cell therapy with existing treatments could potentially improve outcomes for patients with anti-GBM disease and ANCA-associated crescentic GN, addressing the urgent need for more effective and targeted therapies.

## 15. Anti-Glomerular Basement Membrane Glomerulonephritis, ANCA-Associated Crescentic Glomerulonephritis, and Renal Progenitors

Renal progenitor cells, particularly those localized to the inner surface of Bowman’s capsule, play a significant role in the formation of hyperplastic lesions in podocytopathies and crescentic GN. These progenitors can differentiate into podocytes, contributing to kidney regeneration after injury. However, dysregulated proliferation of these cells can lead to pathological conditions. In crescentic GN, massive hyperplasia of parietal epithelial cells, which include RPCs, results in crescent formation and rapid kidney-function decline [100,159]. *In vitro* studies demonstrated that angiotensin II induces the production of SDF-1 in podocytes, subsequently activating glomerular parietal epithelial cells and therefore RPCs. These findings suggest that treatment with angiotensin-converting enzyme inhibitors could limit PEC activation and reduce crescent formation in extra capillary GN [160].

Further studies have shown that crescents originate from the clonal expansion of a single RPC clone, similar to monoclonal diseases in hematopoietic stem cells. This clonal expansion is driven by factors such as TGF-β, which induces RPCs to produce extracellular matrix and express proliferation markers like Ki67 [100,159]. In conditions like FSGS, albuminuria inhibits RPC differentiation into podocytes, leading to the proliferation of immature podocytes and hyperplastic lesions [161,162] (Figure 1). Therapeutic interventions targeting these mechanisms, such as histone deacetylase inhibitors (HDACi) like Panobinostat, have shown promise in reducing crescent formation and promoting RPC differentiation into podocytes, thereby restoring the glomerular filtration barrier and improving kidney function [159,163]. Additionally, markers like CD133 and nestin have been identified in hyperplastic podocytes and crescents, further supporting the involvement of RPCs in these lesions [164,165].

Overall, the evidence underscores the critical role of RPCs in the pathogenesis of hyperplastic lesions in podocytopathies and crescentic GN, highlighting potential therapeutic targets to mitigate these conditions.

## 16. Limitations in Using Stem-Cell Therapy for Renal Diseases

Stem-cell therapy holds significant promise for treating renal diseases due to its regenerative capabilities and potential to improve kidney function. However, there are several limitations and challenges associated with the use of stem cells in renal disease treatment. These limitations range from biological and technical issues to safety and ethical concerns, which need to be addressed to fully harness the potential of stem cell therapies.

Specifically, engraftment and differentiation challenges should be considered. Stem cells often face poor engraftment and limited differentiation in the renal environment, particularly in conditions like diabetic nephropathy. The diabetic microenvironment can hinder stem-cell integration and lead to differentiation into unwanted cell lineages, posing a significant challenge to effective therapy. Moreover, there is a risk of malignant transformation or genetic aberrations in stem cells, which can lead to tumor formation. This is a critical safety concern that needs to be addressed through rigorous preclinical testing and monitoring.

In particular, for the kidney tissues, there is heterogeneity and structural complexity that can constitute a further barrier to overcome and that complicates the application of stem-cell therapies. The diverse histological subtypes and genetic modifications in such renal diseases require highly personalized and targeted approaches, which are still under development. In addition, although MSCs have low immunogenicity, the risk of immune rejection remains, especially in the context of kidney transplantation. The immunomodulatory properties of stem cells are promising, but their long-term safety and effectiveness in clinical settings are still under investigation.

Furthermore, there is a need for standardized protocols for the culture, isolation, and purification of stem cells and their derivatives, such as extracellular vesicles. This standardization is crucial for ensuring consistent quality and efficacy in clinical applications. Lastly, the high cost of stem-cell therapies and the complexity of their production and administration can limit accessibility for patients. Developing cost-effective and scalable solutions is essential for widespread adoption [39].

Despite these limitations, stem-cell therapy remains a promising avenue for renal disease treatment. Advances in stem-cell technology, such as the development of urine-derived stem cells and stem-cell-derived organoids, offer new opportunities for overcoming current challenges. These problems may be solved by improving the innate regenerative capacities of renal progenitors, strategically increasing the suitability and application of these cells, already present in our kidneys, to treat renal diseases. These innovations could lead to more effective and personalized treatments for renal diseases in the future. However, ongoing research and clinical trials are necessary to address the existing limitations and to ensure the safe and effective use of stem cells in renal disease therapy.

## 17. Conclusions

Extensive research in the regenerative medicine field has demonstrated the immense potential of harnessing the regenerative capabilities of stem and progenitor cells to significantly improve outcomes for patients with a wide range of kidney diseases (Table 1).

As discussed in the previous paragraphs, the use of stem-cell secretomes, including soluble factors and extracellular vesicles, as cell-free therapies is also being investigated as a novel approach to induce reparative processes without the risks associated with cell transplantation. Overall, the integration of advanced technologies into stem-cell research and therapy offers a promising avenue for developing more effective treatments for kidney diseases, potentially transforming the landscape of renal medicine and improving patient outcomes. Moreover, by deepening our understanding of the pivotal role these cells play in the intricate pathophysiology of renal disorders, we can pave the way for the development of innovative, targeted treatment approaches that aim to not only restore kidney function but also effectively mitigate the progression of these debilitating conditions. As discussed, renal progenitors may play an underestimated and important role in renal diseases. When RPCs become dysfunctional, they can exacerbate some kinds of renal pathologies, whereas in other cases, they can contribute to damage repair and disease remission. This is crucial, as current treatment options are often suboptimal, and the prognosis for many patients remains poor. In the future, RPCs may be appropriately stimulated to exert their immunomodulatory and reparative functions. Exploring the utilization of stem cells to harness their regenerative potential, immunomodulatory properties, and capacity to mitigate the devastating consequences of autoimmune-mediated glomerular injury may hold the key to improving outcomes and potentially altering the natural history of these devastating kidney diseases.

Unlocking the therapeutic potential of these cells could revolutionize the way we approach the treatment and care of individuals with complex renal disorders, offering new avenues for restoring organ function and ultimately improving their overall quality of life.

## Figures and Tables

**Table 1:** Table summarizing the role of stem cells in IgAN, LN, DN, C3 glomerulopathy, FSGS, IMN, and anti-GBM crescentic GN.

DISEASE	MOLECULAR MECHANISMS OF THE PATHOLOGY	STUDIED STEM CELLS	STUDIED ORGANISM	POSSIBLE MOLECULAR MECHANISMS OF STEM CELLS	POSSIBLE MOLECULAR MECHANISMS OF RENAL PROGENITOR CELLS
Immunoglobulin A nephropathy (IgAN)	Galactose-deficient IgA1 leading to the formation of immune complexes in the kidney.	ADSCs	Mouse [28]	Reduction of fibrotic and inflammatory molecules (TGF-β1, PAI-1, and MCP-1). Decrease of Th1 cytokine activity Shift to Th2 responses	Endothelial mesenchymal transition (EndMT) Inhibition Inflammatory mediators like MCP-1, IL-6, IL-8, and C3.
			Human—phase 1 study of adult patients with refractory IgA nephropathy [29]	Study for the evaluation of safety and tolerability of the treatment	
		VSELs	Human [31]	Improving tissue regeneration	
		EPCs	Rat [39]	HIF-1a and MCP-1 down-regulation	
Lupus Nephritis (LN)	Formation of immunecomplexes (ICs) in the glomeruli, resulting in kidney inflammation, injury, and scarring	MSCs	LN patients [49,50,51] Mouse [51]	Immunomodulatory effects Modulation of cytokines secretion (IL-10, IL-17, TGF-β, TNF-α), GM-CSF, and HMGB-1	Notch pathway activation Immunomodulatory properties
Diabetic nephropathy (DN)	Insulin resistance, genetics, hyperglycemia, and an autoimmune process	MSCs	Rhesus macaque model of DN [71]	Reduction of *SGLT2* expression on RTECs Modulation of inflammation via the Lipoxin A4 LXA4-ALX/FPR2 axis Anti-fibrotic effect	Dysregulation of: Notch pathway WNT/β-catenin Renin-angiotensin-aldosterone system TLR2 signaling
			SD rat [72])		
		BM-MSCs	Mouse [2,75]	EGF and IL-10 serum levels increase Systemic inflammation reduction HGF and MCP-1 secretion	
		BM-MSCs	SD rat [73]		
		MSC-derived EVs	Mouse [77]	Tissue repair Immunomodulation (paracrine mechanism) Anti-apoptotic effect (miRNA-let-7a) Autophagy (inhibition of mTOR signaling pathway) Anti-fibrotic effect	
		ADSCs	Rat [78]	Klotho activation WNT/β-catenin, oxidative stress, and p38-MAPK signaling pathways inhibition	
		ADSCs derived EVs	Mouse [81]	Transport of EGF miRNA cargo (MiR-26a-5p) Anti-apoptosis NF-κB inactivation (by targeting TLR4)	
		USCs	Mouse [90]	TLR 2 and TLR 4 signaling pathways Immunomodulatory effect TGF-β1, α-SMA, collagen I, HSP-47 mRNA reduction E-cadherin, BMP-7 increase	
		USC-derived EVs	Rat [99,100]	Carrier of TGF-β1, angiopoietin, and BMP7→anti-apoptotic effect on podocytes	
C3 glomerulopathy	Dysregulation of the alternative complement pathway may be due to autoantibodies stabilizing C3/C5 convertases or genetic mutations in complement components or inhibitors. The result is a predominant deposition of complement C3 in renal tissue.	Autologous Hematopoietic Stem Cell Transplant	Patients with C3 Glomerulonephiritis secondary to monoclonal gammopathy [117,118]		TLR2 stimulation and expression of high local levels of C3
		hAECs	Mouse [119]	Production of human FH; prevention of complement activation and glomerular deposition of C3	
Focal Segmental Glomerulosclerosis (FSGS)	Podocyte injury, capillary hypertension, and dysregulation of signaling molecules like TGFβ and angiotensin	UCB-MSCs	Mouse [127]	Anti-fibrotic Podocyte and basement membrane recovery	CSF-1R upregulation; activation of the ERK pathway and profibrotic phenotype
		BM-MSCs	Rat [126]	Downregulation *TIMP-1* and upregulation *MMP9* Anti-inflammatory effect (downregulation of *IL-6* and *TNF-α*) Reduction of Col-IV and FN Downregulation of *p38MAPK* and P-CREB	
Idiopathic Membranous Nephropathy (IMN)	Autoantibodies targeting specific podocyte proteins such as PLA2R, THSD7A, and NELL-1. Autoimmune reactions lead to deposits of IgG and complement components in the GBM.	hESC-IMRCs	Rat [138]	Immunomodulatory functions Shift of T lymphocytes from Th1 to Th2 Activation of Treg cells IL-10 production Reduction of IL-17, TNF-α, and IgG and C3 deposits	Immunomodulatory capacity Ability to secrete the C3 complement factor
Anti-Glomerular Basement Membrane (GBM) antibody-associated GN	Polyclonal IgG antibodies targeting the NC1 domain of type IV collagen, peroxidasin and laminin-521. Autoreactive T cells contributing to enhance B cell function and antibody production.	ADSCs	Rat [152]	Reduced immune cell recruitment (CD8+ T cells, CD68+ macrophages) Release of PGE2 and IL-6; phenotypic switching of glomerular macrophages to immunoregulatory CD163+ cells	Production of extracellular matrix; expression of Ki67
		h-GSTM2-MSCs	Mouse [153]	Immunomodulatory effect Increase of antioxidant catalase and glutathione peroxidase 1 genes	
		hMSCs	Rat [151]	Downregulation of proinflammatory cytokine levels Promotion of Th2 and Tregs	
		MSC-CM	Rat [155]	Anti-inflammatory Immunomodulatory effects (IL-10 increase) MCP-1 induction of M2-type macrophage polarization	

The table includes the molecular mechanisms responsible for the above-mentioned renal diseases, together with stem-cell-based therapies, their effects on the studied organisms, and the possible role of renal progenitor cells in these diseases. The abbreviations listed are defined as follows; ADSC: adipose tissue-derived stem cells; VSELs: very small embryonic-like stem cells; EPCs: endothelial progenitor cells; MSCs: mesenchymal stem cells; BM-MSC: bone-marrow-derived mesenchymal stem cells; EVs: extracellular vesicles; USCs urine-derived stem cells; HSCs: hematopoietic stem cells; hAECs: human amniotic epithelial cells; UCB-MSCs: umbilical cord blood-derived mesenchymal stromal cells; hESC-IMRCs: human embryonic stem-cell-derived immunity and matrix regulatory cells; h-GSTM2-MSCs: glutathione S-transferase Mu 2 mesenchymal stem cell; hMSCs: human mesenchymal stem cells; MSC-CM: mesenchymal stem-cells-conditioned medium.
